# Exploring the Gut Microbiome in Combat Sports: A Systematic Scoping Review

**DOI:** 10.3390/sports14010019

**Published:** 2026-01-04

**Authors:** Junior Carlone, Carlo Rossi, Antonino Bianco, Patrik Drid, Attilio Parisi, Alessio Fasano

**Affiliations:** 1 Department of Neurosciences, Biomedicine and Movement, University of Verona, 37134 Verona, Italy; 2 Department of Movement, Human and Health Sciences, University of Rome “Foro Italico”, 00135 Rome, Italy; 3 Division of Pediatric Gastroenterology and Nutrition, Mass General for Children and Harvard Medical School, Boston, MA 02114, USA; 4 Sport and Exercise Research Unit, Department of Psychology, Educational Science and Human Movement, University of Palermo, 90144 Palermo, Italy; 5 Faculty of Sport and Physical Education, University of Novi Sad, 21000 Novi Sad, Serbia; 6 Department of Nutrition, Harvard T.H. Chan School of Public Health, Boston, MA 02115, USA; 7 European Biomedical Research Institute of Salerno, 84125 Salerno, Italy

**Keywords:** gut microbiome, combat sports, athletic performance, sports nutrition, prebiotics, probiotics

## Abstract

The gut microbiota represents a complex microbial ecosystem with the potential to influence athletic performance, energy metabolism, inflammatory responses, and recovery capacity in athletes. However, the specific relationship between microbiota and performance in combat sport athletes remains poorly characterized. This scoping review systematically maps current evidence on gut microbiota-combat sports performance relationships, identifies microbial response patterns to training and competition, evaluates nutritional prebiotic and probiotic interventions, and highlights methodological gaps to guide future research. Following the PRISMA-ScR framework, 8 studies were identified, which included 247 elite and high-level athletes, comprising 169 males and 78 females, with sample sizes ranging from 12 to 53 across wrestling, mixed martial arts, martial arts, judo, and taekwondo. Associations were observed between gut microbiota characteristics and training intensity, competition level, weight management, and pre-competition psychological states. Limited taxonomic consistency was observed across studies, with most bacterial genera appearing in a single investigation, precluding the identification of robust sport-specific microbial signatures. Preliminary trials demonstrated improvements in gastrointestinal symptoms, aerobic performance, and psychological fatigue with prebiotic and probiotic interventions. However, small sample sizes and methodological heterogeneity across studies limit generalizability and preclude definitive conclusions regarding the role of gut microbiome in combat sports performance.

## 1. Introduction

Combat sports (CS), including wrestling, mixed martial arts (MMA), martial arts, judo, boxing, and taekwondo, require a combination of strength, power, endurance, and agility, presenting distinctive physiological and metabolic demands on athletes [1]. Combat sport athletes (CSA) are subjected to intense training and competition regimens characterized by high-intensity intermittent exercise alternating between aerobic and anaerobic demands, weight control practices, frequent competitions, and psychological stress, factors that can influence gut microbiota composition and function [2,3,4]. These sport-specific characteristics could contribute to distinctive gut microbiota profiles in CSA compared with athletes from other disciplines, with variability in microbiota composition influenced by training and competition demands, dietary strategies, and travel-related stressors [5,6,7]. Despite its potential role in modulating athletic performance and training adaptations, gut microbiota remains relatively underexplored in CS, with specific physiological characteristics providing a valuable model for investigating microbiome responses to training and competition [5,8,9,10,11].

Recent investigations have explored the relationship between microbiome and various aspects of performance in CS, including weight management, post-exercise recovery, inflammatory responses, and pre-competition psychological states. Emerging evidence suggests that distinct microbiota compositions contribute to athletic performance by influencing inflammatory markers, energy metabolism, and immune function [9,12,13]. Understanding these microbiota-performance interactions offers opportunities to optimize athletic outcomes through microbiota-targeted approaches [9,14,15,16,17,18].

However, CSA are exposed to challenges, including weight control practices, that can compromise the integrity of gut microbiota [8,19,20,21,22,23,24]. Rapid weight loss (RWL) is prevalent in disciplines such as boxing, wrestling, judo, and MMA, where athletes often aim to lose at least 5% of their body weight within 3–7 days before official weigh-ins, typically employing methods such as extreme dehydration, calorie reduction, and increased exercise [25,26,27,28,29]. This practice can alter microbial composition, with detrimental effects on gut health and athletic performance [30]. Evidence suggests that microbial diversity can be reduced in athletes following extreme dietary restrictions, leading to increased inflammatory markers and decreased immune function, highlighting the critical interplay between weight management practices and microbiome health [9,31,32,33,34,35,36,37,38,39].

Given these challenges, probiotic supplementation has been shown to have beneficial effects on microbiota composition and aerobic performance in athletes [10,12,40,41,42,43,44]. Growing evidence suggests that gut microbiome plays a role in optimizing performance, providing a rationale for manipulating microbiota composition through targeted nutritional strategies. Prebiotic and probiotic interventions represent promising approaches for enhancing gut health and competitive outcomes in CSA [10,12,45,46,47,48]. Despite these insights, methodological heterogeneity and limited sample sizes remain a challenge in current research [49].

This systematic scoping review aims to identify available evidence on the relationship between CS performance and microbiome, with a particular focus on performance-related biomarkers, and to identify recurring patterns in microbiota responses to training and competition. It further analyzes direct correlations between specific microbial profiles and quantitative performance indicators, such as power, endurance, and recovery times. Finally, it evaluates the effectiveness of nutritional and dietary supplementation strategies for modulating gut microbiota to enhance athletic performance, while highlighting methodological and knowledge gaps that may limit the current understanding of the microbiome’s role in CS.

## 2. Materials and Methods

This scoping review was conducted following the PRISMA-ScR (Preferred Reporting Items for Systematic Reviews and Meta-Analyses Extension for Scoping Reviews) methodological framework [50] (Supplementory File S1). The protocol was developed a priori to systematically guide the research and analysis process. The current scoping review was registered with the Open Science Framework (OSF) under the following DOI: 10.17605/OSF.IO/2G48C, identifier 2G48C.

### 2.1. Study Design

The choice to conduct a systematic scoping review stems from the heterogeneity of methodological approaches in available studies examining gut microbiome-CS relationships, the emerging nature of this research field with limited standardized evidence, the need to map existing evidence across diverse CS disciplines, the complexity of microbiome-performance interactions requiring exploratory analysis, and the importance of identifying significant methodological gaps to guide future research directions.

### 2.2. Eligibility Criteria

The inclusion criteria were structured following the Population, Concept, and Context (PCC) framework, including elite and high-level CSA (P), gut microbiota analysis, nutritional interventions, and prebiotic, probiotic, postbiotic, and synbiotic supplementation (C) and athletic performance, weight management, and competition outcomes (C) [50]. Eligible studies included original research, such as randomized controlled trials (RCTs) and observational studies (including cross-sectional, prospective, and retrospective studies), as well as systematic and narrative reviews published in the English language. CS disciplines included wrestling, MMA, judo, taekwondo, boxing, kickboxing, Brazilian jiu-jitsu, and other martial arts practiced at competitive levels. Elite and high-level athletes were defined either according to the criteria reported in the included studies or, when these were not available, based on their competition level. Elite athletes competed at the highest national or international levels, while high-level athletes engaged in structured training and competed at regional or national levels. The exclusion criteria were precisely defined, including studies on non-athletic populations, recreational or non-competitive athletes, studies with incomplete microbiota data, studies conducted exclusively on animal models, and publications in languages other than English.

### 2.3. Research Strategy

To identify relevant studies, a systematic review of the existing literature on the topics of microbiome and CS was conducted. Studies examining the relationship between microbiota and athletic performance, pre-competition anxiety, weight management, and prebiotic or probiotic interventions in CSA. Systematic database searches were conducted in the PubMed, Scopus and Web of Science databases using MeSH terms and keywords related to three main domains: (“Gastrointestinal Microbiome” OR “Gut Microbiome” OR “Gut Microbiota” OR “Intestinal Microbiota”) AND (“Exercise” OR “Physical Activity” OR “Physical Activities” OR “Sports” OR “Athletic” OR “Athletics”) AND (“Judo” OR “Mixed Martial Arts” OR “MMA” OR “Jiu Jitsu” OR “Brazilian Jiu Jitsu” OR “BJJ” OR “Taekwondo” OR “Wrestling” OR “Boxing” OR “Kickboxing” OR “Muay Thai” OR “Karate” OR “Sambo” OR “Kung Fu” OR “Wushu” OR “Capoeira” OR “Kendo” OR “Martial Arts” OR “Combat Sports”). The search was limited to peer-reviewed publications in English, published up to 8 November 2025, excluding grey literature.

### 2.4. Selection Process, Data Extraction and Analysis

The study selection process followed a systematic approach, including an initial screening of titles and abstracts, full-text evaluation of potentially eligible articles, and exclusion of duplicate articles. J.C. and C.R. independently conducted the selection process and data extraction, with A.F. and A.P. consulted to resolve any discrepancies.

Particular attention was given to studies specifically analyzing the relationship between gut microbiota and CSA. Data were extracted using a standardized form, which included study characteristics, population, design, duration, microbiota analysis methodology, training protocols, nutritional interventions, primary and secondary outcomes, main results, and limitations.

### 2.5. Quality Assessment

In accordance with scoping review methodology, we did not employ standardized quality assessment tools, as the primary objective was to map available evidence and identify knowledge gaps rather than evaluate intervention effectiveness [50]. This methodological approach aligns with the exploratory nature of research in the emerging field of microbiome in CS. For the 8 included studies, we systematically assessed key methodological characteristics, including populations and sample sizes, presence of a control group, intervention duration, dropout rates, microbiota analysis methodologies, and main limitations (Table S1). This evaluation revealed that 3 studies employed RCTs designs [51,52,53], 3 utilized cross-sectional observational approaches [54,55,56], and 2 used a prospective design focusing [57,58]. Sample sizes ranged from 12 to 53 participants, with intervention durations of 4 to 8 weeks for the controlled studies. Several methodological considerations emerged across the included studies. Six of eight investigations relied on 16S rRNA sequencing for microbiota analysis, which offers practical advantages but provides limited taxonomic resolution compared to shotgun metagenomic approaches [51,53,54,55,56,57], while one study employed shallow shotgun sequencing [52]. Only four studies complemented their 16S analysis with metabolomic profiling or functional predictions, providing more comprehensive insights into microbiota functionality [51,54,56,57]. One study focused exclusively on fecal organic acid analysis without a full characterization of the microbiota [58].

Inherent challenges in conducting research with elite CSA contributed to observed methodological limitations, including predominantly small sample sizes, limited availability of appropriate control groups, and logistical constraints imposed by competition schedules, training periodization demands, and weight management practices. These factors, combined with the specialized nature of CS populations and ethical constraints regarding experimental interventions during competitive periods, explain the predominance of observational studies and relatively short intervention durations in the available literature.

## 3. Results

**Figure 1:**
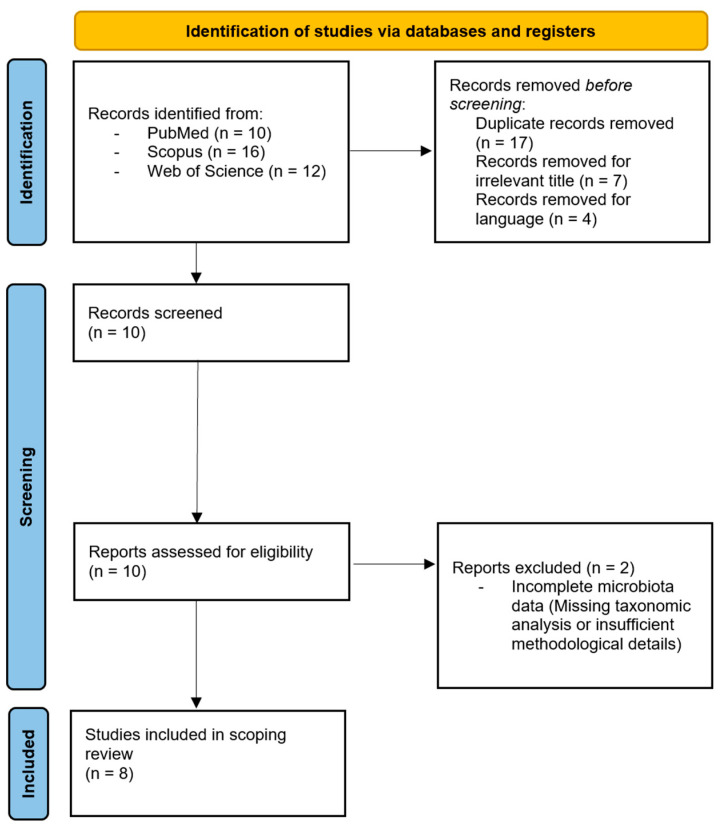
The PRISMA Flow Chart.

The analysis included eight studies examining the relationship between gut microbiota and performance in CSA, including 3 studies on wrestling, 1 on MMA, 1 on wushu martial arts, 1 on judo, and 1 on taekwondo. No eligible systematic or narrative reviews were identified. All eight included studies were original research articles (Figure 1).

### 3.1. Study Characteristics

A total of 247 CSA were included in this systematic scoping review, comprising diverse populations across multiple CS disciplines: wrestlers (n = 77; 65 male, 12 female), MMA (n = 23 male), judokas (n = 20 male), wushu martial arts (n = 28; 13 male, 15 female), and taekwondo (n = 99; 48 male, 51 female). This diverse sample enabled comprehensive analysis across various CS modalities and sexes. The athletic level varied across studies, although they consistently involved competitive populations, including elite and high-level athletes. This distinction is critical, as elite and high-level athletes exhibit fundamentally different microbiota profiles compared to sedentary individuals or recreational athletes, which can be attributed to differences in training volume, dietary patterns, travel demands, and physiological adaptations [6,7]. The duration of interventions ranged from several weeks to two months. Microbiota analysis methodologies mainly included 16S rRNA sequencing, shotgun metagenomics, and mass spectrometry for metabolomic analysis (Table 1 and Table S1).

### 3.2. Microbiome, Athletic Performance and Anxiety

Analysis of the collected data reveals a relationship between gut microbiota composition and various aspects of performance in CS. In particular, observational data suggest that athletes with better performance may exhibit distinctive microbial profiles, though causal relationships cannot always be established. In the study by Przewłócka et al. on high-level MMA athletes, it was observed that combined supplementation of probiotics *Bifidobacterium lactis* W51, *Levilactobacillus brevis* W63, *Lactobacillus acidophilus* W22, *Bifidobacterium bifidum* W23, *Lactococcus lactis* W58, and Vitamin D3 for 4 weeks was associated with an increase in β-diversity of gut microbiota with increases in *Bacteroides*, *Peptostreptococcaceae*, *Roseburia inulinivorans*, and *Prevotella* and a reduction in *Lachnospiraceae* [52]. Microbiota changes were associated with an improvement in aerobic performance, as indicated by an increase in time to exhaustion during the maximal oxygen consumption (VO_2max_) test compared to the group receiving Vitamin D3 supplementation alone [52]. Beyond supplementation interventions, observational studies have identified sport-specific microbial signatures. The examination of gut microbiota in 53 high-level wrestling athletes has identified a possible distinctive microbial pattern characteristic of this athletic population [55]. The study analysis identified *Psychrobacter* as the most discriminating genus for wrestlers [55]. In particular, the observation that specific microbial taxa in wrestlers showed a positive correlation with anaerobic performance parameters, including upper limb anaerobic workload and measurements of maximum and average anaerobic power, was noted [55].

A comparison between competitive levels within martial arts was conducted in 28 elite wushu athletes [56]. Higher-level athletes demonstrated significantly greater gut microbial diversity compared to lower-level athletes, as assessed by the Shannon and Simpson diversity [56]. The genera *Parabacteroides*, *Phascolarctobacterium*, *Oscillibacter*, *and Bilophila* were enriched in higher-level athletes, whereas *Megasphaera* was more abundant in lower-level athletes. Notably, *Parabacteroides* abundance showed a positive correlation with weekly exercise load, with higher-level athletes engaging in more training compared to lower-level athletes [56]. Functional analysis revealed an enhanced metabolic capacity for histidine and carbohydrate metabolism in higher-level athletes [56].

Fu et al. further explored the connection between pre-competition anxiety, microbiota, and performance in 6 male and 6 female elite wrestlers, revealing significant associations between various anxiety indicators, microbial profiles, and sports performance [54]. Wrestlers with better performance showed significantly higher levels of self-efficacy and somatic state anxiety, but lower levels of individual failure anxiety and sports competition anxiety compared to those with lower performance [54]. The group with better performance also presented a more diversified and abundant gut microbiota. Differential metabolites in the optimal performance group were enriched in metabolic pathways related to caffeine metabolism, lipopolysaccharide biosynthesis, vascular endothelial growth factor (VEGF), and mammalian target of rapamycin (mTOR) signalling pathways, suggesting a potential physiological basis for performance differences [54].

### 3.3. Weight Control and Microbiome

Recent investigations in elite wrestlers of both sexes have reported significant variations in gut microbiota composition among CSA according to their pre-competition weight management outcomes [57]. Wrestlers with more effective weight control, with a difference from target weight of less than (<) 2 kg, showed a more appropriate nutritional structure and greater adaptability to training compared to the group with less effective weight control, with a difference from target weight of more than (>) 2 kg [57]. Metabolomic analysis identified divergent metabolites between the two groups, with specific metabolites negatively correlated with high carbohydrate and low protein intake [57]. In particular, the group with a difference from target weight of <2 kg had significantly higher relative protein energy intake and lower fat and carbohydrate intake compared to the group with a difference from target weight of >2 kg [57]. Wrestlers in the group with a difference from target weight of >2 kg also showed greater training adaptation problems, evidenced by the higher detection rate of white blood cells, occult blood, and proteins in urinary analyses, while these indicators were absent in the <2 kg group [57]. At the microbial level, the abundance of *Oscillospiraceae* UCG 002/003, *Eubacterium siraeum*, and *Lachnospiraceae* was positively correlated with the relative energy intake of carbohydrates, while the abundance of *Streptococcus* was negatively correlated with carbohydrate energy intake [57]. These microbial differences were also reflected in functional predictions, with significant differences in prolactin signalling pathway, riboflavin metabolism, epithelial cell signalling in *Helicobacter pylori* infection, and selenium compound pathways, suggesting an impact on athletes’ antioxidant and anti-inflammatory capacities [57]. The differential metabolites were functionally enriched in processes related to lipid and amino acid metabolism. There were 371 divergent metabolites in the <2 kg group when compared with the >2 kg group, comprising 141 upregulated and 230 downregulated metabolites [57].

### 3.4. Fecal Organic Acid Profile and Performance

Research conducted on 20 judokas stratified by competitive level has examined the impact of the fecal organic acid profile on the response to Tabata-type high-intensity training (T-HIIT) [58]. Results indicate that T-HIIT significantly improved the specialized endurance of judokas, as measured by an increase in repetitions in the Uchikomi shuttle run test. A negative correlation was observed between fecal succinic acid concentration and improvements in physical fitness tests [58]. High-level judokas had higher fecal concentrations of acetic and propionic acid and tended to have lower concentrations of succinic acid compared to other athletes [58]. Moreover, the study identified a significant correlation between stool consistency, assessed using the Bristol stool form scale (BSFS), and succinic acid concentrations. Judokas with higher succinic acid concentrations tended to have softer stools, suggesting a possible association with gut dysbiosis [58]. Fecal succinic acid concentrations were negatively correlated with performance improvements after T-HIIT training, specifically in countermovement jumps and pull-ups. High-level judokas showed higher concentrations of acetic and propionic acid, two short-chain fatty acids (SCFAs) with gut barrier integrity and anti-inflammatory effects [58].

### 3.5. Prebiotic and Probiotic Interventions

One recent study investigated the effects of prebiotic supplementation, specifically evaluating konjac glucomannan (KGM) in a double-blind RCT involving 48 elite male taekwondo athletes diagnosed with functional constipation according to Rome IV criteria [51]. Following 8 weeks of intervention with 3 g daily KGM versus placebo (maltodextrin), the KGM group demonstrated significant improvements in gastrointestinal (GI) symptoms assessed through multiple validated instruments: patient assessment of constipation symptoms (PAC-SYM), patient assessment of constipation quality of life (PAC-QoL), bowel movement frequency (BMF), and bowel function index (BFI) [51]. Microbiota analysis revealed increased α-diversity and elevated relative abundances of beneficial taxa, including *Prevotella*-9, *Phascolarctobacterium*, *Lactobacillus*, *Bacteroides*, and *Prevotellaceae* family members, alongside reduced levels of *Alistipes* and *Desulfovibrio* [51]. Functional predictions indicated upregulation of the biotin biosynthesis I and nitrate reduction VI pathways, with downregulation of L-methionine biosynthesis III. These findings suggest that prebiotic dietary fibre interventions may help alleviate functional constipation in CSA through gut microbiota modulation mechanisms [51].

Przewłócka et al. studied the effect of combined supplementation with probiotics (*Bifidobacterium* ssp. and *Lactobacillus* ssp.) and Vitamin D3 in male high-level MMA athletes. Athletes were divided into two groups: one received a combination of probiotics and Vitamin D3, and the other received only Vitamin D3 [52]. Results showed that combined supplementation significantly reduced fecal calprotectin levels. No significant variations were observed in fecal zonulin levels, suggesting that the intervention improved gut inflammation without significantly altering gut barrier permeability [52].

Daily supplementation with probiotic yoghurt containing *Bifidobacterium animalis* ssp. *lactis* BB-12 was administered to 51 high-level female taekwondo athletes for 8 weeks. This intervention appeared to improve recovery from exercise-related psychological fatigue, as assessed by the athlete burnout questionnaire (ABQ), while also influencing gut microbiota composition [53]. The dietary intervention group supplemented with probiotics showed a significant decrease in ABQ scores, whereas no significant change was observed in the control group. The intervention also significantly modified gut microbiota composition, with increases in *Bifidobacteriaceae*, *Bifidobacterium*, *Lactobacillaceae*, and *Lactobacillus* and a decrease in *Escherichia coli* in the probiotic dietary intervention group [53]. Analysis of the predicted function of the microbiota revealed that metabolic pathways of L-arginine I biosynthesis, fatty acid synthesis, oxidation, and L-isoleucine III biosynthesis were significantly more active in the probiotic dietary intervention group compared to the control group [53]. These pathways are known for their role in supporting cognitive function, regulating stress, and improving recovery from fatigue. In particular, it was observed that catecholamine I degradation was significantly reduced in the probiotic dietary intervention group [53]. The absence of an isocaloric control intervention, such as non-probiotic yoghurt or an equivalent protein source, limits the ability to isolate the specific effect of the *Bifidobacterium animalis* ssp. *lactis* BB-12 strain from other nutritional components of the yoghurt matrix.

## 4. Discussion

**Figure 2:**
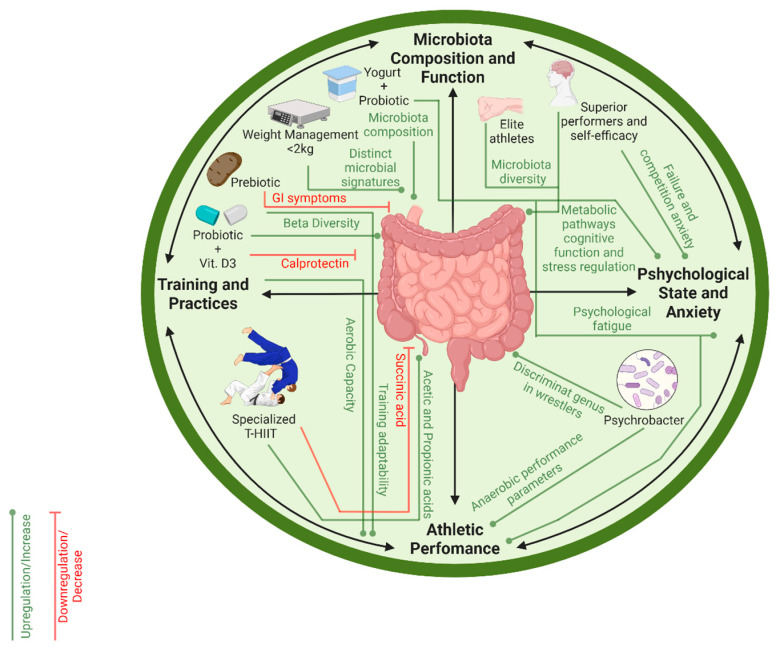
Schematic representation of the microbiota-performance axis in CS. The circular diagram illustrates the multidirectional interactions between intestinal microbial composition, psychological state, and athletic performance. Nutritional interventions, including prebiotics, probiotics, and yoghurt, along with training practices such as T-HIIT and weight management, can modulate the microbiota, influencing both physiological parameters, such as energy metabolism and inflammation, and psychological factors, including pre-competition anxiety and fatigue, with direct effects on performance.

The present analysis of studies examining the microbiome in CSA highlights potential associations between microbial composition and performance-related outcomes, including training adaptations, inflammatory responses, weight management, dietary interventions, and pre-competition psychological states (Figure 2). Although heterogeneity in methodologies and the limited sample sizes constrain generalizability, preliminary evidence supports a possible role of the gut microbiota in optimizing athletic performance through multiple physiological, psychological, and potential metabolic pathways.

### 4.1. Sport-Specific Microbial Adaptations

The eight included studies examining different CSA have reported varied microbial profiles. Given the heterogeneity in study designs, populations, and methodologies, it is challenging to clearly identify sport-specific adaptations. Each observation is derived from a single study examining a unique population, which limits the generalizability of the findings. Specific bacterial genera, such as *Bacteroides*, *Roseburia*, *and Prevotella*, appear across multiple studies, suggesting a possible importance in responses to intense training [52]. These findings suggest a potential functional plasticity of the microbiota in response to the varying metabolic demands of CS. This plasticity may have implications for optimizing performance and recovery. However, without direct comparative studies between different CS or against appropriate control groups, definitive sport-specific adaptations cannot be established.

In high-level female taekwondo athletes, a study revealed that females might be particularly sensitive to microbiome-brain interactions, which could influence exercise-related psychological fatigue [53]. These results, in line with other studies, suggest the need for sex-specific nutritional and training approaches that consider the specificity of the microbial response [59,60]. Furthermore, specialized high-intensity judo training in elite male athletes appears to improve fecal organic acid profiles [58].

### 4.2. Inflammation, Gut Barrier and Weight Loss

A recurring element in the analyzed studies is the relationship between microbiota composition, inflammatory markers, GI disorders, and intestinal permeability in CSA. Prebiotic supplementation with KGM has demonstrated a possible improvement in constipation symptoms in elite taekwondo male athletes through mechanisms involving gut microbiota modulation, with increases in beneficial taxa combined with upregulation of biotin biosynthesis and nitrate reduction pathways that may contribute to enhanced intestinal barrier function and colonic motility [51]. These findings underscore the potential importance of dietary fibre intake in CSA and suggest that GI health optimization through microbiota-targeted nutritional strategies requires further investigation in this population [32].

Probiotic supplementation, particularly when combined with Vitamin D3, has been shown to have a possible effect on reducing inflammatory biomarkers such as calprotectin and improving gut barrier integrity [53]. This modulation of inflammation and gut barrier function represents a potential mechanism through which the microbiota can positively influence performance and recovery in sports that require intense training loads and frequent fluctuations in weight.

Evidence also suggests that gut dysbiosis induced by aggressive RWL in male and female CSA could potentially contribute to systemic inflammation, thereby possibly compromising performance and recovery. Conversely, a balanced microbiota might help mitigate these negative effects [57,61,62]. However, given the limitations of currently available methodologies for measuring intestinal permeability biomarkers, conclusions regarding effects on intestinal barrier function must be interpreted with caution, as these commercial kits may detect proteins other than zonulin and exhibit poor correlation with gold-standard permeability measurements. Intervention studies have demonstrated the direct effects of probiotic supplementation on reducing inflammatory biomarkers, such as calprotectin. In contrast, observational correlations between microbiota composition and inflammatory markers in weight management contexts require cautious interpretation due to potential confounding factors, including diet, training history, genetics, and environmental factors [52,57].

Based on current evidence, gut microbiota appears to influence CS performance through systemic inflammation, energy metabolism, and the production of bioactive metabolites that affect muscle and neural function, as well as gut–brain interactions that impact psychological states. These mechanisms remain to be validated in sex-specific CSA.

### 4.3. Psychological State, Performance and Microbiome

A particularly relevant aspect that emerged from the analysis is the correlation between microbiota composition, pre-competition psychological states, and performance in CSA. Studies suggest that specific microbial profiles may be associated with more favourable pre-competition anxiety states and better overall performance. In line with previously published findings, investigations have demonstrated a correlation between specific bacterial taxa and various aspects of pre-competition anxiety in a small cohort of male and female wrestlers with high awareness and self-efficacy [57,63,64]. Additionally, supplementation with *Bifidobacterium animalis* ssp. *lactis* BB-12 has been shown to improve psychological fatigue scores in high-level female taekwondo athletes through specific alterations of the microbiota and upregulation of metabolic pathways linked to cognitive function and stress management [53]. This, in line with the literature, suggests a possible gut–brain axis that could influence both psychological and physical aspects of performance in CS [65,66]. The mechanism may be particularly relevant in these disciplines, where management of mental fatigue and psychological pressure is a crucial component of overall performance. These findings highlight the potential link between gut microbiota composition, psychological state, and athletic performance in CS, warranting further investigation into a microbiota-brain-performance axis, including studies with appropriate control groups.

### 4.4. Sex-Specific Microbiome in Combat Sports

Within the 247 CSA included in this systematic review (169 males and 78 females), only one study specifically examined a substantial cohort of female athletes, highlighting the underrepresentation of female-focused research. Zhu et al. investigated 51 high-level female taekwondo athletes, suggesting that females may be particularly sensitive to microbiota-brain interactions, with potential implications for managing exercise-related psychological fatigue [53]. The remaining seven studies either included predominantly male athletes or both sexes without performing detailed sex-stratified analyses of microbiota composition and performance outcomes. Notably, Fu et al., despite analyzing a small cohort of six elite female wrestlers, reported that females may exhibit distinct adaptations related to weight management [57]. Similarly, Li et al. observed in a multi-sport cohort that included 53 high-level male wrestling athletes that female athletes had higher Shannon microbial diversity compared to non-athletes, and that gut microbial composition could be stratified by exercise intensity in both sexes [55].

This analytical gap represents a limitation, as emerging evidence, although still preliminary, suggests potential sex differences in gut microbiota composition and functionality. Sex-specific physiological factors, including hormonal fluctuations across the menstrual cycle, differences in gut transit time, variations in immune responses, and distinct metabolic profiles, have been shown to potentially influence microbial diversity and taxonomic composition [67,68,69]. Females appear to exhibit different relative abundances compared to males, with hormonal variations potentially affecting microbial metabolism and host-microbe interactions [70].

In athletic populations specifically, these sex differences could interact with training adaptations, nutritional requirements, and recovery processes in ways that remain poorly understood in CS contexts [71]. Weight management practices common in CS may also affect males and females differently through sex-specific metabolic and hormonal responses. RWL protocols have been shown to alter gut microbiota composition, but whether these alterations differ by sex in CSA remains unexplored [25,29,30]. Similarly, the three intervention studies examining prebiotic and probiotic supplementation showed promising results, although they did not evaluate whether treatment effects varied by sex, highlighting an opportunity for future research on sex-dependent responses to dietary interventions targeting the microbiota [51,52].

Sex-stratified analyses can help identify distinct microbial profiles, evaluate whether microbiota-targeted nutritional interventions exert similar effects in males and females, and examine interactions among hormones, training, and competition.

### 4.5. Dietary Assessment and Standardization in Gut Microbiome Research

A key methodological consideration involves dietary assessment and control, as diet represents a primary determinant of gut microbiota composition [72,73]. While several studies have incorporated dietary assessment tools, such as food frequency questionnaires (FFQs), dietary recalls, or surveys of dietary habits, it is essential to distinguish between dietary assessment and dietary standardization, and to consider the potential biases associated with the recall period [74]. Accurately assessing dietary patterns alone does not fully prevent dietary variation from influencing microbiota-related outcomes, highlighting the importance and the daily challenge of implementing controlled dietary protocols for researchers and athletes, while underscoring the need for such protocols in future studies. In this context, the dietary protocols across the included studies ranged from detailed assessments to the absence of dietary control, reflecting the methodological challenges inherent to research with athletes.

This represents a limitation, as dietary composition, particularly macronutrient distribution, fibre intake, and consumption of fermented foods, has a profound effect on microbial diversity, taxonomic composition, and functional capacity [75,76]. Dietary interventions can induce rapid shifts in gut microbiota composition, with studies demonstrating substantial microbial community changes within 24 to 48 h following dietary modifications [75,76,77]. Variations in protein, carbohydrate, and fat intake can alter gut microbiota profiles within days, potentially confounding associations between training variables and microbial outcomes [77,78,79]. Furthermore, CSA often employ extreme nutritional strategies during weight management phases, including severe caloric restriction, altered macronutrient ratios, and temporary elimination of specific food groups, all of which can substantially alter gut microbiota composition [79].

Intervention studies examining the effects of prebiotic and probiotic supplementation on gut microbiota composition have shown promising results. Even when dietary standardization protocols were not consistently reported, findings suggest that bioactive components in food, together with the supplemented strains, contribute substantially to the observed outcomes. The implementation of isocaloric controls or prospective dietary monitoring can further clarify the specific effects of probiotic strains. Similarly, observational studies comparing athletes of different competitive levels or weight management strategies provide valuable insights into how dietary patterns and training interact to influence gut microbiota composition [9,80,81]. Incorporating dietary variables as covariates allows for a clearer understanding of the relative influence of training and competitive stress versus diet [82].

Taken together, integrating dietary assessment and standardization enables more precise and individualized analyses of microbiota in CSA. This approach emphasizes the interplay between diet, training, and weight management, supporting the development of tailored strategies to optimize health, performance, and microbiota modulation.

### 4.6. Strengths and Limitations

This scoping review presents several key strengths. The systematic search strategy, following PRISMA-ScR guidelines, ensured thorough identification and synthesis of peer-reviewed evidence in this emerging field. The eight included studies comprised 247 participants across multiple CS disciplines, providing preliminary evidence for understanding the relationships between microbiota and performance. The sample included a considerable proportion of female athletes, offering initial insights into this underrepresented population, although sex-specific analyses remain an area requiring further investigation.

Several key limitations must be acknowledged. The limited number of available studies limits the strength of evidence. Many relationships are supported by single studies, including the correlation between fecal organic acid profiles and pre-competition anxiety, requiring independent confirmation. Significant methodological heterogeneity across studies, including variability in study design, microbiome analysis methods, dietary assessment and control protocols, weight management practices, and training interventions, limits direct comparisons between CS disciplines and intervention approaches [83,84]. Furthermore, the predominantly short-term nature of interventions limits understanding of long-term performance effects. Additionally, the temporal stability of microbiota changes after interventions remains poorly characterized [16]. These methodological constraints limit definitive comparative conclusions across different CS contexts.

### 4.7. Practical Applications and Future Developments

Preliminary evidence suggests that prebiotic and probiotic interventions may support GI health, recovery, and performance in CSA. Practical applications include personalized nutritional strategies, tailoring macronutrient and fibre intake based on individual microbiota profiles, periodic dietary and microbiota monitoring to optimize training and competition adaptation, and weight management [85,86,87]. Additionally, educating athletes and coaching staff on dietary strategies and equipment hygiene may help reduce pathogen transmission risks, particularly relevant in contact-based CS, and support overall athlete health [88].

Future research should focus on defining sport-specific microbial profiles across different CS and identifying targeted prebiotic and probiotic interventions through well-powered, longitudinal, randomized trials. Studies should incorporate accurate dietary assessment and standardization, appropriate methodological controls, and evaluate sex-specific responses, seasonal variations, and phases of weight management. The long-term effects of microbiota modulation on health and performance require further investigation.

## 5. Conclusions

This systematic scoping review identified eight emerging studies in CS, providing evidence linking gut microbiota composition to performance outcomes. Limited taxonomic consistency was observed across studies, with most bacterial genera appearing in a single investigation, precluding the identification of robust sport-specific microbial signatures. Associations were observed between gut microbiota characteristics and training intensity, competition level, weight management, and pre-competition psychological states, although causal relationships remain to be established. Preliminary trials have shown improvements in GI symptoms, aerobic performance, and psychological fatigue with prebiotic and probiotic interventions. However, it is unclear whether these changes reflect sport-specific adaptations or general responses to supplementation.

Current evidence is limited by small sample sizes, limited sex-stratified analyses and dietary control, single-study observations, and the predominant use of 16S rRNA sequencing. Future studies should incorporate larger samples, standardized methodologies, systematic dietary assessments, and standardization, as well as sex-stratified analyses and longitudinal designs. The use of 16S rRNA sequencing to characterize microbial composition and shotgun metagenomics to assess functional potential and metabolic capacity will provide evidence-based support for interventions.

## Figures and Tables

**Table 1:** Characteristics of included studies.

Study	Population	Age	Study Design	Intervention/Protocol	Dietary Assessment Tool	Primary Outcomes	Main Findings
Zhu et al. (2025) [51]	Elite Taekwondo Athletes (48 Male)	22.8 ± 3.3 Years	Double-blind RCT	DG: 3 g KGM daily for 8 weeks; CG: 3 g maltodextrin (Placebo) daily for 8 weeks	Semi-quantitative Food Frequency Questionnaire	Gastrointestinal symptoms and gut microbiota composition	Significant improvements observed only in DG: Improved gastrointestinal symptoms, PAC-SYM, PAC-QoL, BMF and BFI. Increased α-diversity and elevated abundances of *Prevotella*_9, *Phascolarctobacterium*, *Lactobacillus*, *Bacteroides*, *Prevotellaceae*. Reduced *Alistipes* and *Desulfovibrio*. Upregulation of Biotin Biosynthesis I, Nitrate Reduction VI and downregulation of L-methionine Biosynthesis III pathways
Fu et al. (2024a) [54]	12 Elite Wrestling Athletes (6 Male and 6 Female)	20.0 ± 1.7 Years	Cross-sectional. Observational	Shared diet and training aimed at weight loss	N/A	Pre-competition anxiety, gut microbiota and metabolites	Better performers had more diverse gut microbiota. Lower competition anxiety in high-performance groups. Metabolites in high performance group enriched in metabolism pathways
Fu et al. (2024b) [57]	12 Elite Wrestling Athletes (6 Male and 6 Female)	20.1 ± 2.0 Years	Observational, Prospective	Dietary and training adaptations for weight control	24 h Dietary recall × 3 days	Psychological fatigue, gut microbiota and metabolites	Superior weight control linked to better nutrition. Better training adaptation in <2 kg weight loss group. Distinct gut microbiota and metabolic pathways
Yoshikawa et al. (2024) [58]	High-level Judo Athletes (10 + 10 Male)	19.5 ± 1.2 Years	Prospective	Specialized Tabata T-HIIT every 2 days for 4 weeks	N/A	Uchikomi shuttle run performance, fecal organic acids	Improved specialized endurance in judoka. Negative correlation between fecal succinic acid and performance improvements. High-level competitors had different organic acid profiles
Li et al. (2023) [55]	High-level Wrestling Athletes (53 male)	16 ± 4 Years	Multi-cohort; Cross-sectional	Sport-specific training	Generic Dietary Questionnaire (20 measurements)	Gut microbiota, inflammatory markers and body composition	Sport-specific gut microbiota profiles, *Prevotella*-driven subgroup linked to inflammation. Sex dependent effects of exercise intensity
Zhu et al. (2023) [53]	High-level Taekwondo Athletes (51 Female)	22.3 ± 0.6 Years	RCT	DK: 250 mL probiotic yoghurt (*Bifidobacterium animalis* ssp. *lactis* BB-12) daily for 8 weeks; CK: routine training without dietary intervention	Dietary Habits Questionnaire + Yoghurt intake monitoring	Psychological fatigue and gut microbiota	Significant improvements observed only in DK: Improved psychological fatigue recovery. Increased beneficial gut bacteria. Enhanced metabolic pathways
Przewłócka et al. (2023) [52]	High-level MMA Athletes (23 Male)	26.0 ± 4.0 Years	RCT	Group 1: Combined probiotics *(Bifidobacterium lactis* W51, *Levilactobacillus brevis* W63, *Lactobacillus acidophilus* W22, *Bifidobacterium bifidum* W23, *Lactococcus lactis* W58) + Vitamin D3; Group 2: Vitamin D3 only for 4 weeks	3-day Food interview + Food Frequency Questionnaire + Supplements survey	Aerobic performance, gut microbiota composition, intestinal permeability and inflammatory markers	Significant improvements observed only in Group 1: Significantly extended time to exhaustion in VO_2max_ test. Increased β-diversity of gut microbiota. Reduced fecal calprotectin and increased *Bacteroides*, *Roseburia* and *Prevotella*
Liang et al. (2019) [56]	28 High-level Wushu Martial Arts Athletes (13 male and 15 female)	20/24 Years	Cross-sectional. Observational	Sport-specific training compared between competitive levels	Feces Pre-collection Questionnaire (3-month recall)	Gut microbiota diversity, taxonomic composition, functional metabolism	Higher-level athletes showed significantly higher α-diversity. Enhanced metabolic pathways for histidine and carbohydrate metabolism than lower-level athletes; *Parabacteroides* correlated with exercise load; *Parabacteroides*, *Phascolarctobacterium*, *Oscillibacter* and *Bilophila* enriched in higher-level athletes; *Megasphaera* is abundant in lower-level athletes.

RCT = Randomized Controlled Trial; DG = Diet intervention group; KGM = Konjac Glucomannan; CG = Control Group; PAC-SYM = Patient Assessment of Constipation Symptoms; PAC-QoL = Patient Assessment of Constipation Quality of Life; BMF = Bowel Movement Frequency; BFI = Bowel Function Index; N/A = Not Applicable; h = hours; T-HIIT = Tabata High-Intensity Interval Training; DK = dietary intervention group; CK = control group; MMA = Mixed Martial Arts; VO_2max_ = maximal oxygen consumption.

## Data Availability

All data generated or analyzed during this study are included in this published article. The data extraction forms and PRISMA-ScR checklist are available from the corresponding author upon reasonable request.

## Supplementary Materials

The following supporting information can be downloaded at: https://www.mdpi.com/article/10.3390/sports14010019/s1, File S1: Preferred Reporting Items for Systematic reviews and Meta-Analyses extension for Scoping Reviews (PRISMA-ScR) Checklist; Table S1: Methodological Characteristics Assessment for Original Studies. Reference [89] is cited in the Supplementary Materials.

## References

[1] Barley O.R., Chapman D.W., Guppy S.N., Abbiss C.R. (2019). Considerations When Assessing Endurance in Combat Sport Athletes. Front. Physiol..

[2] Rossi C., Roklicer R., Tubic T., Bianco A., Gentile A., Manojlovic M., Maksimovic N., Trivic T., Drid P. (2022). The Role of Psychological Factors in Judo: A Systematic Review. Int. J. Environ. Res. Public Health.

[3] Fontana F., Longhi G., Tarracchini C., Mancabelli L., Lugli G.A., Alessandri G., Turroni F., Milani C., Ventura M. (2023). The human gut microbiome of athletes: Metagenomic and metabolic insights. Microbiome.

[4] Clark A., Mach N. (2016). Exercise-induced stress behavior, gut-microbiota-brain axis and diet: A systematic review for athletes. J. Int. Soc. Sports Nutr..

[5] O’Donovan C.M., Madigan S.M., Garcia-Perez I., Rankin A., O’ Sullivan O., Cotter P.D. (2020). Distinct microbiome composition and metabolome exists across subgroups of elite Irish athletes. J. Sci. Med. Sport.

[6] Barton W., Penney N.C., Cronin O., Garcia-Perez I., Molloy M.G., Holmes E., Shanahan F., Cotter P.D., O’Sullivan O. (2018). The microbiome of professional athletes differs from that of more sedentary subjects in composition and particularly at the functional metabolic level. Gut.

[7] Fasano A. (2025). The Physiology of Hunger. N. Engl. J. Med..

[8] Fasano A. (2012). Zonulin, regulation of tight junctions, and autoimmune diseases. Ann. N. Y. Acad. Sci..

[9] Mohr A.E., Jäger R., Carpenter K.C., Kerksick C.M., Purpura M., Townsend J.R., West N.P., Black K., Gleeson M., Pyne D.B. (2020). The athletic gut microbiota. J. Int. Soc. Sports Nutr..

[10] Patel B.K., Patel K.H., Lee C.N., Moochhala S. (2024). Intestinal Microbiota Interventions to Enhance Athletic Performance—A Review. Int. J. Mol. Sci..

[11] Carlone J., Lista M., Romagnoli R., Sgrò P., Piacentini M.F., Di Luigi L. (2023). The role of the hormonal profile of constitutional biotypes in the training process. Med. Sport.

[12] Jarrett H., Medlin S., Morehen J.C. (2025). The Role of the Gut Microbiome and Probiotics in Sports Performance: A Narrative Review Update. Nutrients.

[13] Przewłócka K., Folwarski M., Kaźmierczak-Siedlecka K., Skonieczna-Żydecka K., Kaczor J.J. (2020). Gut-Muscle AxisExists and May Affect Skeletal Muscle Adaptation to Training. Nutrients.

[14] Rankin A., O’Donovan C., Madigan S.M., O’Sullivan O., Cotter P.D. (2017). ‘Microbes in sport’-The potential role of the gut microbiota in athlete health and performance. Br. J. Sports Med..

[15] Rinninella E., Raoul P., Cintoni M., Franceschi F., Miggiano G.A.D., Gasbarrini A., Mele M.C. (2019). What is the Healthy Gut Microbiota Composition? A Changing Ecosystem across Age, Environment, Diet, and Diseases. Microorganisms.

[16] Carlone J., Giampaoli S., Alladio E., Rosellini G., Barni F., Salata E., Parisi A., Fasano A., Tessitore A. (2025). Dynamic stability of gut microbiota in elite volleyball athletes: Microbial adaptations during training, competition and recovery. Front. Sports Act. Living.

[17] Mousavinasab F., Karimi R., Taheri S., Ahmadvand F., Sanaaee S., Najafi S., Halvaii M.S., Haghgoo A., Zamany M., Majidpoor J. (2023). Microbiome modulation in inflammatory diseases: Progress to microbiome genetic engineering. Cancer Cell Int..

[18] Zheng D., Liwinski T., Elinav E. (2020). Interaction between microbiota and immunity in health and disease. Cell Res..

[19] Li J., O’Toole P.W. (2024). Disease-associated microbiome signature species in the gut. PNAS Nexus.

[20] Vargas A., Robinson B.L., Houston K., Vilela Sangay A.R., Saadeh M., D’Souza S., Johnson D.A. (2025). Gut microbiota-derived metabolites and chronic inflammatory diseases. Explor. Med..

[21] Heimer M., Teschler M., Schmitz B., Mooren F.C. (2022). Health Benefits of Probiotics in Sport and Exercise—Non-existent or a Matter of Heterogeneity? A Systematic Review. Front. Nutr..

[22] Keohane D.M., Woods T., O’Connor P., Underwood S., Cronin O., Whiston R., O’Sullivan O., Cotter P., Shanahan F., Molloy M.G.M. (2019). Four men in a boat: Ultra-endurance exercise alters the gut microbiome. J. Sci. Med. Sport.

[23] da Rocha A.L., Teixeira G.R., Pinto A.P., de Morais G.P., Oliveira L.D.C., de Vicente L.G., da Silva L.E.C.M., Pauli J.R., Cintra D.E., Ropelle E.R. (2018). Excessive training induces molecular signs of pathologic cardiac hypertrophy. J. Cell Physiol..

[24] John G.K., Wang L., Nanavati J., Twose C., Singh R., Mullin G. (2018). Dietary Alteration of the Gut Microbiome and Its Impact on Weight and Fat Mass: A Systematic Review and Meta-Analysis. Genes.

[25] Zhong Y., Song Y., Artioli G.G., Gee T.I., French D.N., Zheng H., Lyu M., Li Y. (2024). The Practice of Weight Loss in Combat Sports Athletes: A Systematic Review. Nutrients.

[26] Milovančev A., Ilić A., Miljković T., Petrović M., Stojšić Milosavljević A., Roklicer R., Trivic T., Manojlovic M., Rossi C., Bianco A. (2024). Cardiac biomarkers alterations in rapid weight loss and high-intensity training in judo athletes: A crossover pilot study. J. Sports Med. Phys. Fit..

[27] Lukic-Sarkanovic M., Roklicer R., Trivic T., Manojlovic M., Gilic B., Milovancev A., Rossi C., Bianco A., Carraro A., Cvjeticanin M. (2024). Acute muscle damage as a metabolic response to rapid weight loss in wrestlers. Biomed. Hum. Kinet..

[28] Roklicer R., Rossi C., Bianco A., Štajer V., Maksimovic N., Manojlovic M., Gilic B., Trivic T., Drid P. (2022). Rapid weight loss coupled with sport-specific training impairs heart rate recovery in Greco-Roman wrestlers. Appl. Sci..

[29] Maksimovic N., Cvjeticanin O., Rossi C., Manojlovic M., Roklicer R., Bianco A., Carraro A., Sekulic D., Milovancev A., Trivic T. (2024). Prevalence of metabolic syndrome and its association with rapid weight loss among former elite combat sports athletes in Serbia. BMC Public Health.

[30] Nechalová L., Bielik V., Hric I., Babicová M., Baranovičová E., Grendár M., Koška J., Penesová A. (2024). Gut microbiota and metabolic responses to a 12-week caloric restriction combined with strength and HIIT training in patients with obesity: A randomized trial. BMC Sports Sci. Med. Rehabil..

[31] Clarke S.F., Murphy E.F., O’Sullivan O., Lucey A.J., Humphreys M., Hogan A., Hayes P., O’Reilly M., Jeffery I.B., Wood-Martin R. (2014). Exercise and associated dietary extremes impact on gut microbial diversity. Gut.

[32] Mancin L., Burke L.M., Rollo I. (2025). Fibre: The Forgotten Carbohydrate in Sports Nutrition Recommendations. Sports Med..

[33] Hughes R.L., Holscher H.D. (2021). Fueling Gut Microbes: A Review of the Interaction between Diet, Exercise, and the Gut Microbiota in Athletes. Adv. Nutr..

[34] Contreras F., Al-Najim W., le Roux C.W. (2024). Health Benefits Beyond the Scale: The Role of Diet and Nutrition During Weight Loss Programmes. Nutrients.

[35] Cheng S.C., Chang C., Chen Y.C., Gojobori T., Chiu P.K. (2025). Human gut microbiome determining athletes’ performance: An insight from genomic analysis. Ecol. Genet. Genom..

[36] Ursell L.K., Metcalf J.L., Parfrey L.W., Knight R. (2012). Defining the human microbiome. Nutr Rev..

[37] Arumugam M., Raes J., Pelletier E., Le Paslier D., Yamada T., Mende D.R., Fernandes G.R., Tap J., Bruls T., Batto J.M. (2011). Enterotypes of the human gut microbiome. Nature.

[38] Fasano A. (2020). All disease begins in the (leaky) gut: Role of zonulin-mediated gut permeability in the pathogenesis of some chronic inflammatory diseases. F1000Res.

[39] Fasano A. (2012). Intestinal permeability and its regulation by zonulin: Diagnostic and therapeutic implications. Clin. Gastroenterol. Hepatol..

[40] Marttinen M., Ala-Jaakkola R., Laitila A., Lehtinen M.J. (2020). Gut Microbiota, Probiotics and Physical Performance in Athletes and Physically Active Individuals. Nutrients.

[41] Jäger R., Mohr A.E., Carpenter K.C., Kerksick C.M., Purpura M., Moussa A., Townsend J.R., Lamprecht M., West N.P., Black K. (2019). International Society of Sports Nutrition Position Stand: Probiotics. J. Int. Soc. Sports Nutr..

[42] Marchesi J.R., Adams D.H., Fava F., Hermes G.D., Hirschfield G.M., Hold G., Quraishi M.N., Kinross J., Smidt H., Tuohy K.M. (2016). The gut microbiota and host health: A new clinical frontier. Gut.

[43] Dallas D.C., Sanctuary M.R., Qu Y., Khajavi S.H., Van Zandt A.E., Dyandra M., Frese S.A., Barile D., German J.B. (2017). Personalizing protein nourishment. Crit. Rev. Food Sci. Nutr..

[44] Donati Zeppa S., Agostini D., Gervasi M., Annibalini G., Amatori S., Ferrini F., Sisti D., Piccoli G., Barbieri E., Sestili P. (2019). Mutual Interactions among Exercise, Sport Supplements and Microbiota. Nutrients.

[45] Turnagöl H.H., Koşar Ş.N., Güzel Y., Aktitiz S., Atakan M.M. (2021). Nutritional Considerations for Injury Prevention and Recovery in Combat Sports. Nutrients.

[46] Xu Y., Zhong F., Zheng X., Lai H.Y., Wu C., Huang C. (2022). Disparity of Gut Microbiota Composition Among Elite Athletes and Young Adults With Different Physical Activity Independent of Dietary Status: A Matching Study. Front. Nutr..

[47] Chen Y., Yang K., Xu M., Zhang Y., Weng X., Luo J., Li Y., Mao Y.H. (2024). Dietary Patterns, Gut Microbiota and Sports Performance in Athletes: A Narrative Review. Nutrients.

[48] Oami T., Shimazui T., Yumoto T., Otani S., Hayashi Y., Coopersmith C.M. (2025). Gut integrity in intensive care: Alterations in host permeability and the microbiome as potential therapeutic targets. J. Intensive Care.

[49] Wu H., Forslund S., Wang Z., Zhao G. (2023). Human Gut Microbiome Researches Over the Last Decade: Current Challenges and Future Directions. Phenomics.

[50] Page M.J., McKenzie J.E., Bossuyt P.M., Boutron I., Hoffmann T.C., Mulrow C.D., Shamseer L., Tetzlaff J.M., Akl E.A., Brennan S.E. (2021). The PRISMA 2020 statement: An updated guideline for reporting systematic reviews. BMJ.

[51] Zhu Y., Chen X., Song G. (2025). Effects of konjac glucomannan on gastrointestinal symptoms and gut microbiota in athletes with functional constipation: A double-blind randomized controlled trial. Eur. J. Nutr..

[52] Przewłócka K., Folwarski M., Kaczmarczyk M., Skonieczna-Żydecka K., Palma J., Bytowska Z.K., Kujach S., Kaczor J.J. (2023). Combined probiotics with vitamin D_3_ supplementation improved aerobic performance and gut microbiome composition in mixed martial arts athletes. Front. Nutr..

[53] Zhu J., Zhu Y., Song G. (2023). Effect of Probiotic Yogurt Supplementation(*Bifidobacterium animalis* ssp. lactis BB-12) on Gut Microbiota of Female Taekwondo Athletes and Its Relationship with Exercise-Related Psychological Fatigue. Microorganisms.

[54] Fu P., Wang C., Zheng S., Qiao L., Gao W., Gong L. (2024). Connection of pre-competition anxiety with gut microbiota and metabolites in wrestlers with varying sports performances based on brain-gut axis theory. BMC Microbiol..

[55] Li Y., Cheng M., Zha Y., Yang K., Tong Y., Wang S., Lu Q., Ning K. (2023). Gut microbiota and inflammation patterns for specialized athletes: A multi-cohort study across different types of sports. mSystems.

[56] Liang R., Zhang S., Peng X., Yang W., Xu Y., Wu P., Chen J., Cai Y., Zhou J. (2019). Characteristics of the gut microbiota in professional martial arts athletes: A comparison between different competition levels. PLoS ONE.

[57] Fu P., Wang C., Zheng S., Gong L. (2024). Differences in gut microbiota and metabolites between wrestlers with varying precompetition weight control effect. Physiol. Genom..

[58] Yoshikawa T., Yokoyama Y., Sakai A., Kuno T., Nimura Y., Matsunami H. (2024). Impact of Fecal Organic Acid Profile Before Training on Athletic Performance Improvement After High-Intensity Interval Training. Int. J. Sports Physiol. Perform..

[59] Scheiman J., Luber J.M., Chavkin T.A., MacDonald T., Tung A., Pham L.D., Wibowo M.C., Wurth R.C., Punthambaker S., Tierney B.T. (2019). Meta-omics analysis of elite athletes identifies a performance-enhancing microbe that functions via lactate metabolism. Nat. Med..

[60] Wohlgemuth K.J., Arieta L.R., Brewer G.J., Hoselton A.L., Gould L.M., Smith-Ryan A.E. (2021). Sex differences and considerations for female specific nutritional strategies: A narrative review. J. Int. Soc. Sports Nutr..

[61] Koutoukidis D.A., Jebb S.A., Zimmerman M., Otunla A., Henry J.A., Ferrey A., Schofield E., Kinton J., Aveyard P., Marchesi J.R. (2022). The association of weight loss with changes in the gut microbiota diversity, composition, and intestinal permeability: A systematic review and meta-analysis. Gut Microbes.

[62] Carías Domínguez A.M., de Jesús Rosa Salazar D., Stefanolo J.P., Cruz Serrano M.C., Casas I.C., Zuluaga Peña J.R. (2025). Intestinal Dysbiosis: Exploring Definition, Associated Symptoms, and Perspectives for a Comprehensive Understanding—A Scoping Review. Probiotics Antimicrob. Proteins.

[63] Kumar A., Pramanik J., Goyal N., Chauhan D., Sivamaruthi B.S., Prajapati B.G., Chaiyasut C. (2023). Gut Microbiota in Anxiety and Depression: Unveiling the Relationships and Management Options. Pharmaceuticals.

[64] Xiong R.G., Li J., Cheng J., Zhou D.D., Wu S.X., Huang S.Y., Saimaiti A., Yang Z.J., Gan R.Y., Li H.B. (2023). The Role of Gut Microbiota in Anxiety, Depression, and Other Mental Disorders as Well as the Protective Effects of Dietary Components. Nutrients.

[65] Wang J., Zhou T., Liu F., Huang Y., Xiao Z., Qian Y., Zhou W. (2023). Influence of gut microbiota on resilience and its possible mechanisms. Int. J. Biol. Sci..

[66] Dalton A., Mermier C., Zuhl M. (2019). Exercise influence on the microbiome-gut-brain axis. Gut Microbes.

[67] Kim Y.S., Unno T., Kim B.Y., Park M.S. (2020). Sex differences in gut microbiota. World J. Men Health.

[68] Markle J.G.M., Frank D.N., Mortin-Toth S., Robertson C.E., Feazel L.M., Rolle-Kampczyk U., von Bergen M., McCoy K.D., Macpherson A.J., Danska J.S. (2013). Sex differences in the gut microbiome drive hormone-dependent regulation of autoimmunity. Science.

[69] Org E., Mehrabian M., Parks B.W., Shipkova P., Liu X., Drake T.A., Lusis A.J. (2016). Sex differences and hormonal effects on gut microbiota composition in mice. Gut Microbes.

[70] Koliada A., Moseiko V., Romanenko M., Lushchak O., Kryzhanovska N., Guryanov V., Vaiserman A. (2021). Sex differences in the phylum-level human gut microbiota composition. BMC Microbiol..

[71] Pugh J.N., Lydon K.M., O’Donovan C.M., O’Sullivan O., Madigan S.M. (2022). More than a gut feeling: What is the role of the gastrointestinal tract in female athlete health?. Eur. J. Sport Sci..

[72] Zmora N., Suez J., Elinav E. (2019). You are what you eat: Diet, health and the gut microbiota. Nat. Rev. Gastroenterol. Hepatol..

[73] Singh R.K., Chang H.-W., Yan D., Lee K.M., Ucmak D., Wong K., Abrouk M., Farahnik B., Nakamura M., Zhu T.H. (2017). Influence of diet on the gut microbiome and implications for human health. J. Transl. Med..

[74] Leeming E.R., Louca P., Gibson R., Menni C., Spector T.D., Le Roy C.I. (2021). The complexities of the diet-microbiome relationship: Advances and perspectives. Genome Med..

[75] Ross F.C., Patangia D., Grimaud G., Lavelle A., Dempsey E.M., Ross R.P., Stanton C. (2024). The interplay between diet and the gut microbiome: Implications for health and disease. Nat. Rev. Microbiol..

[76] Kolodziejczyk A.A., Zheng D., Elinav E. (2019). Diet-microbiota interactions and personalized nutrition. Nat. Rev. Microbiol..

[77] David L.A., Maurice C.F., Carmody R.N., Gootenberg D.B., Button J.E., Wolfe B.E., Ling A.V., Devlin A.S., Varma Y., Fischbach M.A. (2014). Diet rapidly and reproducibly alters the human gut microbiome. Nature.

[78] Gentile C.L., Weir T.L. (2018). The gut microbiota at the intersection of diet and human health. Science.

[79] Cella V., Bimonte V.M., Sabato C., Paoli A., Baldari C., Campanella M., Lenzi A., Ferretti E., Migliaccio S. (2021). Nutrition and physical activity-induced changes in gut microbiota: Possible implications for human health and athletic performance. Foods.

[80] Driuchina A., Isola V., Hulmi J.J., Salmi V.M., Hintikka J., Ahtiainen J.P., Pekkala S. (2025). Unveiling the impact of competition weight loss on gut microbiota: Alterations in diversity, composition, and predicted metabolic functions. J. Int. Soc. Sports Nutr..

[81] Conlon M.A., Bird A.R. (2015). The impact of diet and lifestyle on gut microbiota and human health. Nutrients.

[82] Naderi A., Rothschild J.A., Santos H.O., Hamidvand A., Koozehchian M.S., Ghazzagh A., Berjisian E., Podlogar T. (2025). Nutritional strategies to improve post-exercise recovery and subsequent exercise performance: A narrative review. Sports Med..

[83] Mancin L., Paoli A., Berry S., Gonzalez J.T., Collins A.J., Lizarraga M.A., Mota J.F., Nicola S., Rollo I. (2024). Standardization of gut microbiome analysis in sports. Cell Rep. Med..

[84] Rodriguez J., Hassani Z., Alves Costa Silva C., Betsou F., Carraturo F., Fasano A., Israelsen M., Iyappan A., Krag A., Metwaly A. (2025). State of the art and the future of microbiome-based biomarkers: A multidisciplinary Delphi consensus. Lancet Microbe.

[85] Carlone J., Parisi A., Fasano A. (2025). The performance gut: A key to optimizing performance in high-level athletes: A systematic scoping review. Front. Sports Act. Living.

[86] Carlone J., Giampaoli S., Brancucci A. (2025). Interactions between gut microbiota and brain: Possible effects on sport performance. J. Sci. Sport Exerc..

[87] Gaskell S., Martinez I., Costa R.J.S. (2025). Gut microbiota and exercise: A systematic review of interventions and evidence limitations. Int. J. Sports Med..

[88] Mast E.E., Goodman R.A. (1997). Prevention of infectious disease transmission in sports. Sports Med..

[89] Tricco A.C., Lillie E., Zarin W., O’Brien K.K., Colquhoun H., Levac D., Moher D., Peters M.D.J., Horsley T., Weeks L. (2018). PRISMA Extension for Scoping Reviews (PRISMAScR): Checklist and Explanation. Ann. Intern. Med..

