# Supplementary material for: Exploring the Gut Microbiome in Combat Sports: A Systematic Scoping Review
Source: Sports (Basel). 2026 Jan 4;14(1):19. doi: 10.3390/sports14010019 (PMC12846275; doi:10.3390/sports14010019)
Supplement: Supplementary file 1 [file sports-14-00019-s001.zip › Table S1.pdf]

Exploring The Gut Microbiome in Combat Sports: A Systematic Scoping Review

Table S1 Methodological Characteristics Assessment for Original Studies

Study	Population	Control Group	Duration	Dropout Rate	Microbiota Analysis Methods	Key Methodological Limitations
Zhu et al. (2025) [51]	Elite Taekwondo Athletes (48 M)	Control Group (Placebo)	8 Weeks	8 Subjects	16S rRNA Sequencing and PICRUSt Functional Prediction	Male only Population
Fu et al. (2024a) [54]	12 Elite Wrestling Athletes (6 F and 6 M)	//	Single Timepoint	N/A	16S rRNA Sequencing and Metabolomics	Small Sample Size; Same Participants (Fu et al 2024b); Cross-Sectional Design; No Control Group
Fu et al. (2024b) [57]	12 Elite Wrestling Athletes (6 F and 6 M)	//	Pre-post Weight Control period	N/A	16S rRNA Sequencing and Metabolomics	Small Sample Size; Same Participants (Fu et al 2024a); Short Follow-up; No Control Group
Yoshikawa et al. (2024) [58]	High-level Judo Athletes (10+10 M)	//	4 Weeks	N/A	Fecal organic acid analysis	Male only Population; Limited to Organic Acid Analysis; No Control Group
Li et al. (2023) [55]	Multi-sport Athletes (543 Mixed); High-level Wrestling Athletes (53 M)	Comparisons between sports; No Internal Control Group	Single Timepoint	N/A	16S rRNA Sequencing	Male-only Population; Cross-sectional Design; No Longitudinal Follow-up
Zhu et al. (2023) [53]	High-level Taekwondo Athletes (51 F)	Control Group	8 Weeks	N/A	16S rRNA Sequencing	Female only Populations; Single Probiotic Strain Tested; Sex Population
Przewłócka et al. (2023) [52]	High-level MMA Athletes (23 M)	Control Group	4 Weeks	N/A	Shallow Shotgun Sequencing	Male only Population; Small Sample Size; Short Intervention Duration; Sex Population
Liang et al. (2019) [56]	28 High-level Wushu Martial Arts Athletes (13 M and 15 F)	Internal Comparison	N/A	3 Subjects	16S rRNA Sequencing and PICRUSt Functional Prediction	Cross-Sectional Design; No Longitudinal Data

F = female; M = male; N/A = Not Applicable; MMA = Mixed Martial Arts; ; PICRUSt = Phylogenetic Investigation of Communities by Reconstruction of Unobserved States
